# Lord Lister: IV. Surgery in Pre-Listerian Days

**Published:** 1918-02-16

**Authors:** 


### LORD LISTER.

### IV.—Surgery in Pre-Listerian Days.

THERE are few now left who remember from personal experience the practice of surgery in the pre-Listerian age, and it is difficult for the surgeons of the present generation to realise what the state of surgical practice then was, and what its results were. Every general practitioner now is a surgeon, and very many general practitioners, probably the great majority of them, are experienced surgeons, who will undertake without hesitation and with success most of the operations of surgery. There are some operations of peculiar difficulty and delicacy that are still left to the hands of the pure surgeon who has specialised in the department to which the operations belong; but any well-trained general practitioner will undertake most of the operations of surgery, many of them, it may be said the majority of them, unknown in pre-Listerian days, and not then thought of or to be thought of. To open the peritoneum was to incur deadly danger. To open the knee-joint was regarded, and justly regarded, as certain death. What operations were performed were left to surgeons, to men who practised surgery and nothing else. The general practitioner rarely used the knife. He was afraid of it. The orthodox operations authorised by tradition, by practice, and by the text-books were few. The opportunities for gaining experience in performing them were very few; and consequently they were left to those who devoted their lives to surgery. It was then possible for a man who had taken honours at a great university, and who had filled all the resident posts at a hospital to which a great medical school was attached, to be afraid to open an abscess, so little was the use of the knife practised. To this the present writer can speak from personal observation; and when we remember the consequences that so often, it may be said so generally, followed even a trifling incision, we can well understand the timidity and the reluctance. So few operations were performed, that one operating theatre supplied the needs of a large hospital of 600 or 700 beds, and even in this theatre operations were performed on only one afternoon of the week, and even on this afternoon sometimes there was but one, sometimes there were none at all. An operation for hernia was a rare event, for it was usually fatal. Amputations were the most frequent operations, constituting from one-fifth to one-seventh of the total. "Healing by first intention," that is to say, the immediate healing of the wound without suppuration, which is now the almost invariable rule, was described in the books as a possible result of operation, but in practice it was unknown, and an amputation wound never healed until for weeks it had discharged cataracts of pus; but there was a graver reason for the avoidance of operation if it could be avoided.

For "hospital diseases" followed the use of the knife with deadly pertinacity. Again and again the surgical wards of certain hospitals were compulsorily closed because to enter them as a wounded patient or an operation case was almost certain death. Pyæmia, septicæmia, cellulitis, and erysipelas were endemic in all hospitals of any size; and every now and again hospital gangrene appeared and emptied the wards. The pertinacity with which these diseases clung to hospitals, and the comparative immunity enjoyed by patients in private practice, isolated in their own homes, and especially in country places, could not escape observation, and became at length notorious. There was something, it seemed, in the very air of a hospital, or emanating from the fabric of the walls, that produced these diseases. Erichsen, whose book on surgery was the standard authority, and, indeed, almost the only authority, in the 'seventies of the last century, wrote as follows:—"The town free from infection; the hospital saturated by it, to such an extent as to induce surgeons to recommend their patients not to enter it, to compel them to refrain from operating, and, after every attempt that science and humanity could suggest—every hygienic means employed in vain in the fruitless attempt to eradicate the pestilence from 'the very fabric itself'—to cause the governors, as a last resource, to decide on the demolition of the building and its complete reconstruction, at a great expense, as the only remedy."

This heroic remedy was actually employed in certain cases, but, alas! the new hospitals, built of new materials, were soon as badly infected as the old, or at least, the diseases peculiar to hospitals soon appeared in them and clung to them as they had done to the previous building. It is obvious enough to us, now that Lister has taught us the lesson, that the infection was not in the fabric of the hospital, but in the patients themselves, and that it was carried, and under the system of practice then in use could not but be carried, from one patient to another. It is obvious enough to us now that we have been told, just as it is obvious to us that the earth must rotate, and that the sun cannot be carried round it; just as it is obvious that species of animals and plants must arise from modifications of previous species, and were not each of them specially created in the form in which we see them; but none of these truths that are so obvious to us—now that they have been explained to us—was obvious to those to whom they had not been explained. On the contrary, each of them was received with bitter and determined opposition. The discoverer of each of them was scorned, vilified, and vituperated, as if he had been the vilest and basest of men, and of this treatment Lister had his share—nay, he has his share now. This very week, and for weeks past, he has been vituperated and vilified in the columns of a pro-German weekly paper. So strong was the conviction

☛ Previous articles appeared January 26, page 351, February 2, page 371, and 9, page 393.

**LORD LISTER**—(*continued*).

that the infection was in "the very fabric itself," that Simpson, a successful gynæcologist in his day —the mortality of his ovariotomies was *only* about 36 per cent.—Simpson urged that hospitals should be abolished altogether, and their place supplied by wooden huts, which could be burned from time to time. To some extent this system was actually adopted, and on the Continent some hospitals assumed the dimensions of a small village, being composed of many little buildings, each accommodating but two or three patients, and together occupying many acres of ground. These also were found by experience to be as unhealthy as the old massive hospitals in which hundreds of patients were collected together under one roof.

Meanwhile, while hospitals were being razed to the ground and rebuilt at enormous expense, only to prove as virulent foci of disease as ever, it never occurred to the surgeons that they were themselves the agents that disseminated hospital diseases and caused the dreadful mortality. The operating-table was covered by a leathern cushion that was wiped and washed after an operation, but never changed. The surgeon, before performing an operation, changed his coat for a filthy garment kept for the purpose, and saturated with the blood and other discharges of years of previous operations. The dressers and house surgeons stuffed a dozen or so of soft silk ligatures into their buttonholes, from whence these ligatures were withdrawn to tie the arteries. The nurses attended with bowls full of sponges, wrung out and washed after the last operation, but treated no farther than by washing in tap water. Ordinary tap water was sometimes employed to wash the surface of the wound before it was closed and stitched up, but often this ceremony was omitted. Strangers who were present to watch the operation, and the dressers and students for their instruction, were invited to poke their unwashed fingers into a deep wound in order to satisfy themselves of this point or that. The instruments were wiped, cleaned, and put away in a cupboard lined with baize in readiness for the next operation. If one operation immediately succeeded another, even this measure of cleanliness was omitted. The retractors used for a septic case were used in the next case without any precaution as to cleansing. The present writer has seen a surgeon, who was wont to become extremely agitated over an operation, sweating so profusely that drops of sweat fell from his forehead into the wound he was making. Nor was his forehead the only source of uncleanly drips. He was a snuff-taker, and—but enough has been said to show us, with the knowledge Lister has taught us, why hospitals were such deadly institutions to surgical cases, and especially to operation cases. It must be remembered in addition, however, that when the patient was removed to the ward, his wound was frequently dressed; sometimes by the surgeon, who pretty regularly washed his hands between case and case; sometimes by the house surgeon, who occasionally emulated his chief in this respect; sometimes by a dresser, who never thought of doing so. Add to this that *post-mortem* examinations were made daily in every large hospital; that though the surgeons rarely attended these functions, the house surgeons and dressers attended regularly according to their zeal, and that they would often be summoned from the *post-mortem* room to the receiving-room to take in cases, and sometimes to the wards to attend a case already there; and that nothing beyond a simple and hurried ablution with soap and water intervened between the one and the other; and the causes of the spread of hospital diseases were not far to seek, and need scarcely be attributed to a mysterious miasma attached to "the very fabric itself" of the building. It is all very obvious now, for now we view it in the light of Lister's teaching; but it was not at all obvious then, and Lister's teaching met with much opposition, much ridicule, and much opprobrium.

